# Analysis of Some Empirical Equations for Modeling Sound Absorption of Porous Absorbers

**DOI:** 10.3390/ma19143128

**Published:** 2026-07-21

**Authors:** Valentín Gómez Escobar, Celia Moreno González

**Affiliations:** Departamento de Física Aplicada, Escuela Politécnica, Institute for Sustainable Regional Development (INTERRA), Universidad de Extremadura, Avda. de la Universidad s/n, 10003 Cáceres, Spain; celiamg@unex.es

**Keywords:** porous materials, sound absorption coefficient, theoretical equations, thickness influence, impedance tube

## Abstract

The empirical Delany–Bazley model and several of its subsequent modifications were evaluated to determine their ability to predict the sound absorption coefficient spectra of porous materials. The study considered three porous materials (foam, mineral wool and samples made from recycled cigarette butt) with different thicknesses. Model performance was assessed by comparing theoretical predictions with impedance tube measurements using the root mean square error (RMSE). Although some models yielded lower prediction errors for specific materials and thicknesses, the results showed that sample thickness—and the associated changes in the shape of the sound absorption spectrum—have a greater influence on model performance than the material itself. No single empirical model consistently provided the best agreement over the entire thickness range. These findings suggest that the applicability of Delany–Bazley-type empirical models appears to be more closely related to the shape of the sound absorption spectrum than to the material for which they were originally developed. These findings provide practical guidance for selecting suitable models for both conventional and recycled porous sound absorbers.

## 1. Introduction

Sound-absorbing materials play a key role in the acoustic treatment of rooms and are also widely used in multilayer sound insulation systems.

Porous materials constitute one of the most important classes of sound absorbers. They are characterized by interconnected pores or cavities through which sound waves propagate while losing part of their energy due to thermal and viscous effects. According to their microscopic structure, porous materials can be classified as granular, fibrous, or cellular [1].

Several models have been proposed to predict the acoustic behavior of porous materials. They can be classified as theoretical, microstructural, and phenomenological. A review of some of them can be found elsewhere [2,3]. Apart from their theoretical formulation, the main difference between these models lies in the number of parameters required for the calculation. Because some of the parameters needed by the theoretical models (e.g., porosity, tortuosity, thermal characteristic length, and airflow resistivity) are difficult to determine experimentally, one of the most widely used models is the empirical model proposed by Delany and Bazley [4], which requires only a single parameter: the airflow resistivity of the sample.

Several authors have applied the Delany–Bazley model to compare theoretical predictions with experimental results [5,6,7,8,9,10]. Other authors have proposed modifications to the coefficients of the Delany–Bazley model to adapt it to materials different from the commercial fibrous absorbers (fiberglass and mineral wool) originally considered by Delany and Bazley. Thus, Dunn and Davern [11], Wu [12], and Miki [13] proposed modified versions of the Delany–Bazley model for open-pore polyurethane foams and plastic open-cell foams, and for double-layer porous materials, respectively. Later, Garai and Pompoli [14], Ramis et al. [15], Arenas et al. [16], and Rey et al. [17] proposed further modifications to the original coefficients for polyester-, kenaf-, cellulose-, and sheep wool-based materials, respectively. Recently, Dunne [18] proposed an ‘optimized Delany-Bazley model’ for both synthetic and natural fiber materials.

More recently, other authors have determined optimized coefficients for the Delany-Bazley equation for individual samples [19,20].

In recent years, our research group has investigated the development of a sound-absorbing material produced by recycling used cigarette butts [21,22]. Accordingly, the first objective of the present work is to evaluate the suitability of the original Delany–Bazley equation and several of its modified versions for predicting the acoustic performance of sound-absorbing samples manufactured from recycled cigarette butts.

However, the shape of the sound absorption spectrum is strongly influenced by sample thickness [23,24,25,26]. This raises the question of whether a single empirical model is sufficient to fit the experimental data or whether the predictive capability of these empirical models depends primarily on the type of porous material or on the shape of the sound absorption spectrum itself. Therefore, the second objective of this work is to evaluate the suitability of these equations for samples with different thicknesses and, consequently, different sound absorption spectra.

Finally, to assess the generality of the proposed approach, two additional porous materials, namely a cellular foam and commercial mineral wool, were also investigated.

## 2. Materials and Methods

### 2.1. Instrumentation for the Determination of Acoustic Properties

When a sound wave impinges on a material, part of the incident wave is reflected (*E_r_*), and the remainder of the wave is either dissipated within the material or transmitted through it. Accordingly, the sound absorption coefficient (α) is defined as the ratio of the non-reflected sound wave energy (*E_i_* − *E_r_*) and the incident energy (*E_i_*). Therefore:

(1) $$\alpha =\;\frac{E_{i}-E_{r}}{E_{i}}$$

There are different methods for the determination of the sound absorption coefficient. In this work, the sound absorption coefficient of the samples was determined using an impedance tube, following the two-microphone transfer-function method described in the ISO 10534–2 standard [27].

The measurements were performed using a Brüel & Kjær Impedance Tube Kit (Type 4206, Copenhagen, Denmark), equipped with two one-quarter-inch condenser microphones (Type 4187). Signal acquisition and analysis were carried out using a portable Brüel & Kjær PULSE System (Type 3560-C) with four input channels. Sample holders with diameters of 29 and 100 mm were used, providing valid frequency ranges of 500–6400 Hz and 50–1600 Hz, respectively.

**Figure 1:**
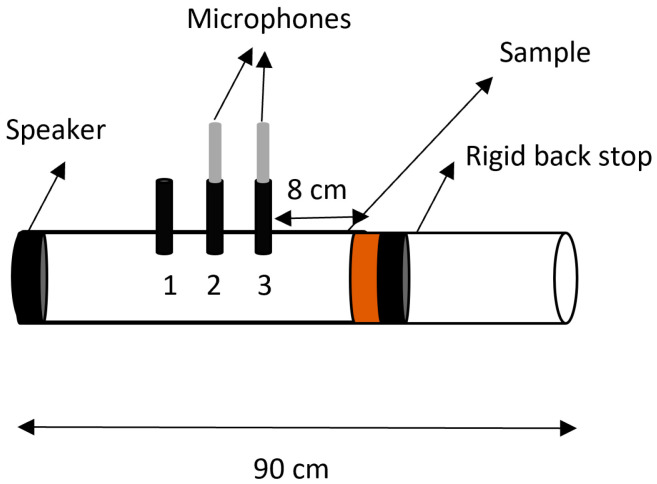
Experimental configuration used for the sound absorption measurements.

The experimental configuration used for the sound absorption measurements is shown in Figure 1.

Airflow resistivity (*σ*) is a fundamental property of porous materials and quantifies the resistance offered by the pore structure to airflow. It is expressed in Pa·s/m^2^ and is related to the airflow resistance (*R*) (defined as the ratio of the pressure drop across the sample to the airflow velocity through it), according to:

(2) $$\sigma =\;\frac{R\cdot A}{d}$$

where *A* is the cross-sectional area of the sample and *d* is the sample thickness.

The airflow resistivity of the samples analyzed in this work was determined using the Ingard and Dear method [28]. The experimental sample configuration inside the impedance tube was the same as described in previous work [29]. The airflow resistivity was determined from the sound pressure levels measured by the two microphones. Following the procedure proposed by Juliá Sanchís [30], the difference between the sound pressure levels at the first minimum of the L_p1_ − L_p2_ spectrum was used to calculate the normalized airflow resistance and, subsequently, the airflow resistivity.

### 2.2. Preparation of Samples

**Figure 2:**
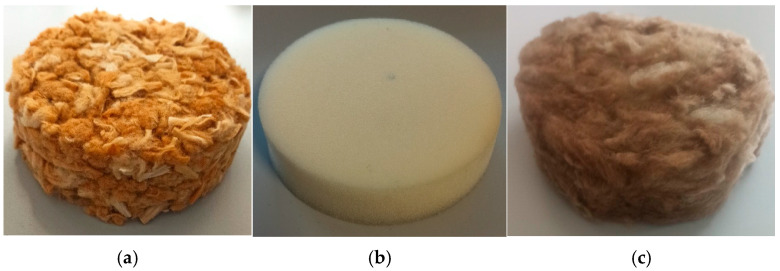
Representative photographs of some of the samples with a 100 mm diameter. (**a**) sample prepared with used cigarette butts; (**b**) foam sample; (**c**) commercial mineral wool sample.

Three different types of materials were considered for the present study: one cellular (foam samples) and two fibrous (commercial mineral wool and samples prepared by disaggregating used cigarette butts). The commercial mineral wool samples were cut directly from commercial panels, whereas the foam samples were supplied with the impedance tube kit as reference materials. The used cigarette butts for the third group of samples were conditioned as described in previous work [29], without chemical cleaning, and subsequently disaggregated using a grinder. Representative photographs of the prepared samples are shown in Figure 2.

Samples were prepared in two sizes, both circular, with diameters of 29 and 100 mm according to the two diameter sizes of the used impedance tube. As mentioned above, previous studies show that the sound absorption coefficient spectrum was influenced by the thickness of the sample. Thus, for each type of sample, several samples of different thicknesses were made. No compression was applied during sample preparation; therefore, differences in density among samples of the same material were minimal. The bulk density of each sample was determined by weighing the samples and calculating their bulk density. The measured bulk density ranged from 63 to 71 kg/m^3^ for butt-derived samples, from 25.9 to 27.1 kg/m^3^ for foam samples, and from 36 to 41 kg/m^3^ for mineral wool samples. The number of samples prepared and their thickness ranges are summarized in Table 1.

The airflow resistivity estimated using the Ingard and Dear method exhibited a marked dependence on sample thickness. Values ranging from 3 to 15 kPa·s/m^2^ were obtained for the foam samples, from 1 to 12 kPa·s/m^2^ for the mineral wool samples, and from 1 to 7 kPa·s/m^2^ for the butts-derived samples. This variation in thickness was consistent with the changes observed in the sound absorption spectra, which will be described below.

### 2.3. Theoretical Equations

The Delany and Bazley model describes the acoustic behavior of porous materials in terms of two complex variables: the characteristic impedance (*Z*_0_) and the propagation coefficient (*γ*) according to the following general equations:

(3) $$Z_{0}=\;\rho_{0}\cdot c_{0}\left(1+C1{\left(\frac{\rho_{0}f}{\sigma }\right)}^{-C2}-j\cdot C3{\left(\frac{\rho_{0}f}{\sigma }\right)}^{-C4}\right)$$

(4) $$\gamma =\frac{\omega }{c_{0}}\left({C5\left(\frac{\rho_{0}f}{\sigma }\right)}^{-C6}+j\;\left(1+C7{\left(\frac{\rho_{0}f}{\sigma }\right)}^{-C8}\right)\right)$$

where the coefficients *C*1 to *C*8 proposed by Delany and Bazley are those indicated in Table 2 (Model M1).

Although some authors prefer to use the complex wave number (*k_c_*) (radians per meter or m^−1^), instead of the propagation coefficient (*γ*) [2,6,31]:

(5) $$k_{c}=\;\frac{\omega }{c_{0}}\left({1+C7\left(\frac{\rho_{0}f}{\sigma }\right)}^{-C8}-j\;{C5\left(\frac{\rho_{0}f}{\sigma }\right)}^{-C6}\right)\;$$

which is equivalent to Equation (4) considering that *k_c_* = *j·γ*.

In their work, Delany and Bazley indicate that the sound absorption coefficient of a sample (dimensionless) can be calculated from the following expression:

(6) $$\alpha =1-\;{\left|\frac{Z-\rho_{0}c}{Z+\rho_{0}c}\right|}^{2}$$

For a rigidly backed layer (as is the case of the described measurements in the impedance tube) of thickness *l*:

*Z* = *Z*_0_·*coth*
*γ*·*l* (7)

In the calculation of the sample impedance [*Z*] (Pa·s/m^2^) for the determination of the sound absorption coefficient, some authors consider *Z*_0_ to be the complex impedance defined by Delany and Bazley [4], while others consider *Z*_0_ to be the product of the speed and the density of air (thus, *Z*_0_ *= ρ*_0_*·c*_0_) [9,16,17]. In the present work, the first assumption was considered, i.e., that *Z*_0_ is the complex impedance defined by Delany and Bazley [the one given in Equation (3)] as it was considered to be the most appropriate to follow the Delany & Bazley work [4].

In this study, ten different theoretical models derived from the initial equations proposed by Delany and Bazley have been considered. Table 2 shows the coefficients of the ten different theoretical models considered in this study. The last of these was proposed by the authors as a reformulation of the theoretical model by Allard and Champoux, adapted to the Delany-Bazley model [32].

**Table 2:** Delany & Bazley derived models considered and their coefficients.

Model		C1	C2	C3	C4	C5	C6	C7	C8	Materials for Which the Model Was Proposed
M1	Delany & Bazley [4]	0.0570	0.7540	0.0870	0.7320	0.1890	0.5950	0.0980	0.7000	Commercial fibrous absorbers
M2	Garai & Pompoli [13]	0.0780	0.6230	0.0740	0.6600	0.1590	0.5710	0.1210	0.5300	Polyester fiber materials
M3	Dunn & Davern [11]	0.1140	0.3690	0.0990	0.7580	0.1680	0.7150	0.1360	0.4910	Open-pore polyurethane foams
M4	Wu [12]	0.2120	0.4550	0.1050	0.6070	0.1630	0.5920	0.1880	0.5440	Plastic open-cell foams
M5	Miki [13]	0.0700	0.6320	0.1070	0.6320	0.1600	0.6180	0.1090	0.6180	Fibrous materials
M6	Ramis et al. [15]	0.0460	0.2550	0.1120	0.9670	0.0600	1.2560	0.0390	0.5410	Kenaf materials
M7	Rey et al. [17]	0.0560	0.3640	0.0160	0.9330	0.1920	0.7860	0.1200	1.1160	Sheep wool materials
M8	Dunne [18]	0.1676	0.5632	0.0860	0.7333	0.2015	0.6159	0.0190	0.9989	Synthetic and natural fiber materials
M9	Arenas et al. [16]	0.6220	0.0890	−0.4810	−0.6140	0.3950	−0.1270	0.5820	−0.0870	Cellulose materials
M10	Oliva & Hongisto [32]	0.0729	0.6623	0.1870	0.5379	0.2880	0.5260	0.0982	0.6850	Wool materials

### 2.4. Model Optimization and Statistical Analysis

To identify the model providing the best agreement with the experimental data, the Root Mean Square Error (RMSE) was used as the goodness-of-fit criterion. The RMSE value was calculated with the following equation:

(8) $$RMSE=\;\sqrt{\sum_{i=1}^{n}\frac{{\left({TV}_{i}-{EV}_{i}\right)}^{2}}{n}}$$

where *EV* and *TV* represent the experimental and theoretical values of the sound absorption coefficient, respectively.

For measurements performed with the 100 mm impedance tube, the RMSE was calculated over the frequency range 100–1600 Hz. Although this tube is valid over the range 50–1600 Hz, the frequency interval between 50 and 100 Hz was excluded because some measurements in this interval exhibited anomalous values. For measurements performed with the 29 mm impedance tube, the RMSE was calculated over the frequency range 500–6400 Hz.

To facilitate comparison among the RMSE values obtained with the different models, the relative increase in RMSE with respect to the minimum RMSE value was calculated:

(9) $$Relative\;RMSE=\;\frac{RMSE\;value-Minimum\;RMSE}{Minimum\;RMSE}\cdot 100$$

## 3. Results and Discussion

### 3.1. Shape of the Sound Absorption Coefficient Spectra

**Figure 3:**
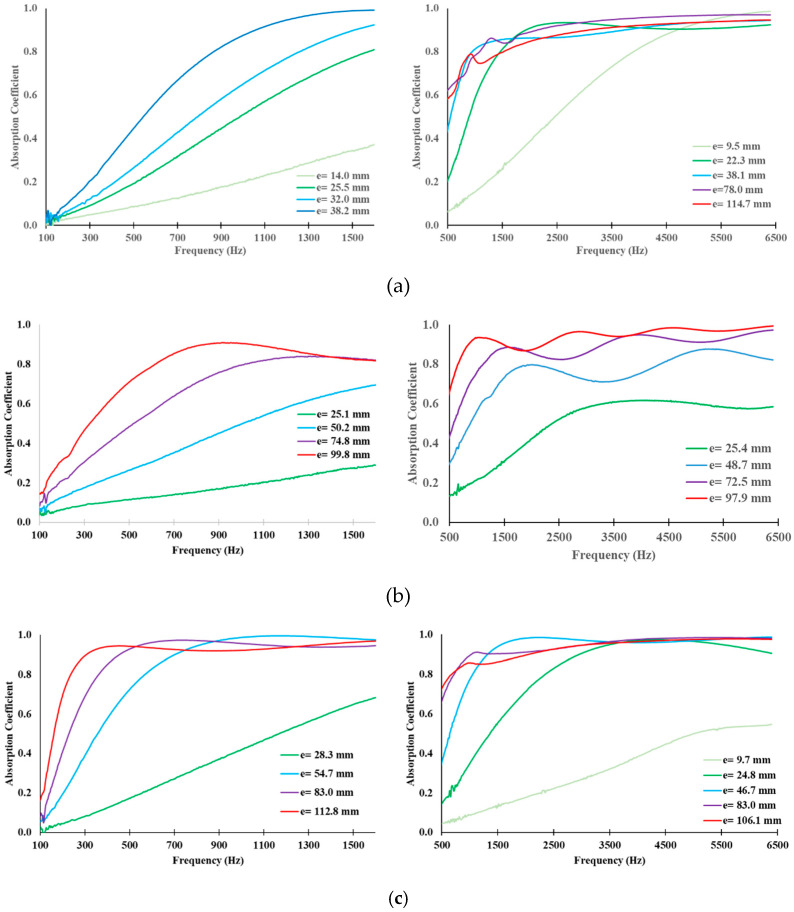
Sound absorption coefficient spectra of representative samples used cigarette butt (**a**), foam (**b**) and mineral wool (**c**) samples with different thicknesses measured using the two impedance tube diameters [left: 100 mm; right: 29 mm].

Figure 3 shows that the shape of the sound absorption spectrum changes markedly with sample thickness for all three materials and for both impedance tube diameters. These results are consistent with those shown in previous studies [26].

It is clear from Figure 3 that increasing the sample thickness affects not only the magnitude of the sound absorption coefficient but also the overall shape of its frequency response. In the case of the 100 mm samples, the spectrum for the thinnest samples is almost linear, increasing the curvature of the spectra with increasing thickness. In the case of the 29 mm samples, the spectra become increasingly complex as thickness increases.

**Figure 4:**
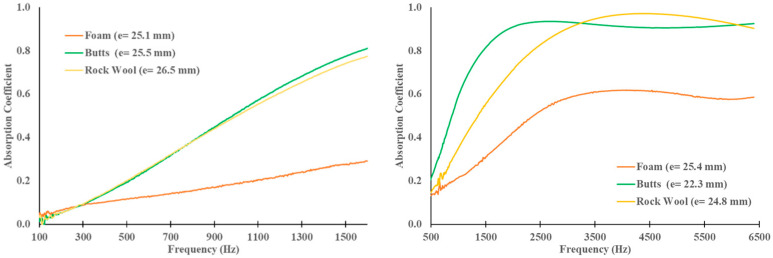
Sound absorption coefficients for some examples of samples of different types with similar thickness, measured using the two impedance tube diameters [(**left**): 100 mm; (**right**): 29 mm].

Figure 4 compares the three materials for similar sample thicknesses. As can be seen, in the 100 mm tube, the cigarette butt and mineral wool samples show similar spectral characteristics. In the 29 mm tube, although some similarity is also observed between the cigarette butt and mineral wool samples, greater variability is observed among the spectra of the types of samples. For both diameters, the spectra of the foam samples show a behavior that is quite different from the others.

These observations raise the question of whether the proposed theoretical models are valid for all thicknesses of the type of sample for which they were proposed and whether they could be applicable to other types of samples.

When sound absorption coefficients are measured using impedance tubes of different diameters, the acquisition system can combine the results into a single broadband spectrum.

However, this procedure is not always appropriate for the samples considered in this study. Unavoidable differences in sample preparation (for example, difficulty in reproducing exactly the same porous structure) may produce discrepancies between the measurements obtained with the 29 and 100 mm impedance tubes. As a result, the overlap region between the two spectra (approximately 1000–2000 Hz) may exhibit slight discontinuities. These differences were observed mainly in butts-derived and mineral wool samples.

Nevertheless, spectra from samples with similar thicknesses were combined and included in the analysis.

**Figure 5:**
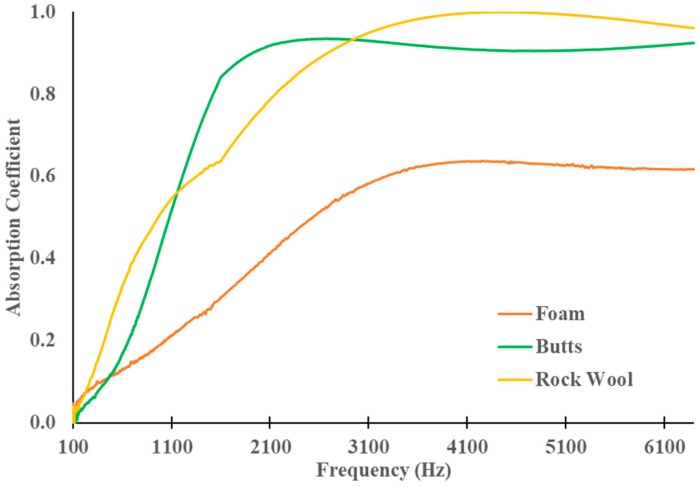
Sound absorption coefficients for combined spectra of the different types of samples with similar thickness (near 25 mm).

Figure 5 presents representative combined spectra for the three materials considered, all with a thickness of approximately 25 mm. A slight discontinuity can be observed in the overlap region, particularly for the mineral wool sample.

### 3.2. Application of the Different Proposed Theoretical Models to the Samples: 100 mm Tube Spectra

The performance of the ten theoretical models was first assessed using the spectra measured with the 100 mm impedance tube (100–1600 Hz). The RMSE values obtained for each sample are summarized in Table 3.

For the recycled cigarette butt samples, model M9 provided the lowest RMSE values for most samples thicker than 25 mm (except one case where model M10 provided the lowest value). For thinner samples, the best agreement was shared among models M7, M8, M9 and M10. For the foam samples, model M6 consistently provided the lowest RMSE values over most of the thickness range, whereas model M2 performed better only for the thickest samples (approximately 100 mm). In the case of mineral wool samples, models M7, M9 and M10 provided the best agreement for thicknesses above 50 mm. For thinner samples, model M10 led to better RMSE values, except in one case, which was model M5.

**Figure 6:**
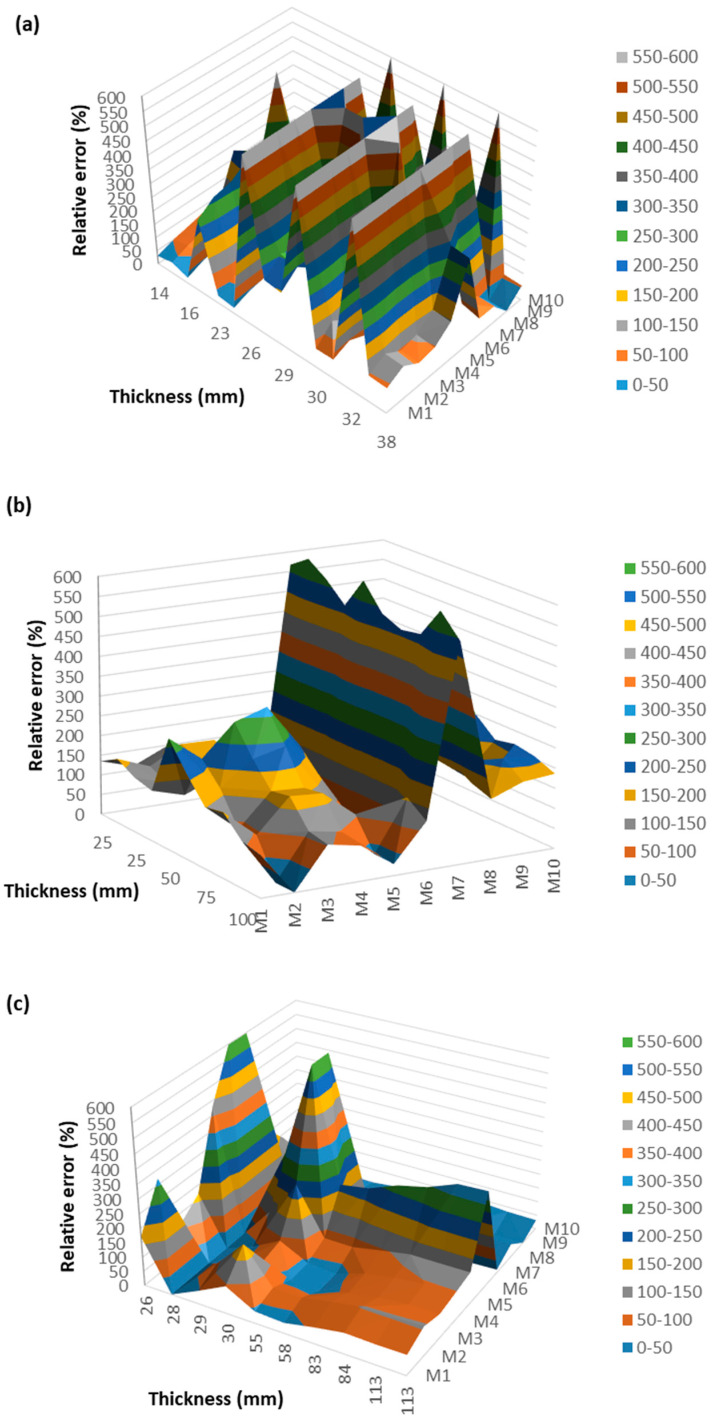
Relative RMSE values for 100 mm samples [(**a**) used cigarette butts; (**b**) foam; (**c**) mineral wool].

To complement the analysis based solely on the minimum RMSE values, the relative RMSE values [Equation (9)] were also calculated, allowing the performance of models with similar fitting accuracy to be compared. The relative RMSE values for all samples and models are presented in Figure 6, whereas the corresponding mean relative RMSE values, grouped according to material type and sample thickness, are summarized in Table 4.

Considering the overall results (row all in Table 4), model M9 provided the lowest mean relative RMSE values for the recycled cigarette butt samples. However, when the influence of sample thickness is taken into account, a more complex behavior is observed. For samples with thicknesses between 10 and 20 mm, model M7 yielded the lowest mean relative RMSE values. This result is consistent with the marked differences observed in the sound absorption spectra of the thinnest samples (Figure 3a). For samples thicker than 20 mm, model M9 consistently provided the best overall agreement, whereas the remaining models generally exhibited mean relative RMSE values exceeding 200%, indicating a substantially poorer fit.

For the foam samples, model M6 yielded the lowest overall mean relative RMSE value (Table 4). The same trend was observed for samples up to approximately 80 mm thick. Only for the thickest samples (around 100 mm) did model M2 outperform the remaining formulations. For all other models, the mean relative RMSE values were generally more than 100% higher than those of the best-performing model.

For the mineral wool samples, model M9 provided the lowest overall mean relative RMSE value and was also the best-performing model in three of the four thickness intervals considered. The only exception corresponded to the 75–100 mm interval, for which model M7 yielded the lowest mean relative RMSE. However, the results for the 25–50 mm interval were strongly influenced by sample MW100-2, for which model M2 produced a substantially lower RMSE than any other formulation. Because this single sample had a pronounced effect on the mean values reported in Table 4, an additional analysis was performed excluding this apparent outlier. The corresponding results are summarized in Table 5.

After excluding sample MW100-2, model M10 provided the lowest overall mean relative RMSE for the mineral wool samples (Table 5). Nevertheless, the optimal model still depended on sample thickness. Model M10 performed best for samples thinner than 50 mm, model M9 yielded the lowest mean relative RMSE values for the 50–75 mm and 100–125 mm intervals, and model M7 was the best-performing model for samples with thicknesses between 75 and 100 mm.

The results presented in this section indicate that foam requires different empirical models from those that best describe recycled cigarette butt and mineral wool samples, which is consistent with its cellular structure compared with the fibrous nature of the other two materials. Furthermore, these results indicate that no single Delany–Bazley-derived formulation provides the best prediction over the entire thickness range. Even for the same material, the optimal model depends strongly on sample thickness.

### 3.3. Application of the Different Proposed Theoretical Models to the Samples: 29 mm Tube Spectra

**Figure 7:**
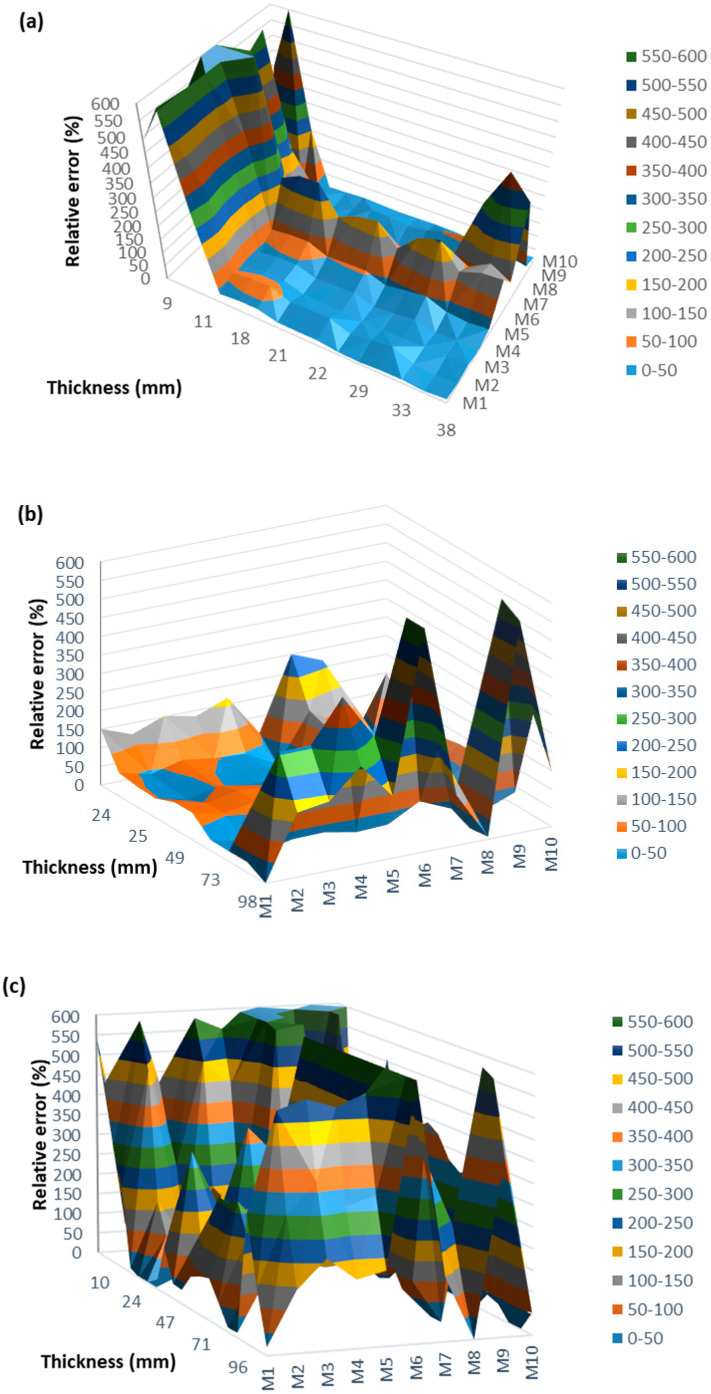
Relative RMSE values for 29 mm samples [(**a**) used cigarette butts; (**b**) foam; (**c**) mineral wool].

The results obtained with the 29 mm impedance tube, corresponding to the frequency range 500–6400 Hz, are summarized in Table 6 and Table 7 and Figure 7. As previously shown in Figure 3, the shape of the sound absorption spectrum changes markedly with sample thickness, suggesting that the suitability of the empirical models may also depend on this parameter.

For the recycled cigarette butt samples, model M9 clearly provided the best agreement for the thinnest individual samples (thicknesses below 11 mm). Moreover, in the 5–20 mm thickness interval, this model is clearly the best, whereas the remaining models yielded relative RMSE values generally exceeding 150%. For thicker samples, however, the differences among most of the models became much less pronounced. Only models M6 and, for the thickest samples, M8 consistently exhibited poorer performance. Within the intermediate thickness range (20–30 mm), models M8 and M10 provided the lowest RMSE values, whereas model M4 performed best for samples between 30 and 40 mm thick. These trends are also evident in Figure 7a.

Considering individual foam samples, model M6 consistently provided the lowest RMSE values for samples up to approximately 50 mm thick. For the thickest samples (around 100 mm), however, models M1 and M8 yielded the best agreement with the experimental measurements. The analysis of the mean relative RMSE values confirms this trend: model M6 performed best for the 20–40 mm and 40–60 mm thickness intervals, whereas models M9 and M8 provided the lowest mean relative RMSE values for the 60–80 mm and 80–100 mm intervals, respectively. In the latter interval, all the remaining formulations, except model M1, exhibited relative RMSE values generally exceeding 125%. Nevertheless, for samples between 25 and 75 mm thick, models M1, M2, M3, M4, M8 and M10 all produced relative RMSE values within 50% of the minimum, indicating that several empirical formulations provide similarly accurate predictions for acoustic foam over this thickness range.

A markedly different behavior was observed for mineral wool samples. Except for the two thinnest samples (approximately 10 mm thick), whose sound absorption spectra differ substantially from those of the thicker samples (Figure 3), model M10 consistently yielded the lowest RMSE values. For the two thinnest samples, model M5 provided the best agreement, whereas model M8 also produced the lowest RMSE values for some samples thicker than 75 mm. Interestingly, model M6, which consistently performed best for the acoustic foam samples, showed one of the poorest performances for mineral wool, highlighting the strong influence of the porous structure on the suitability of the empirical models.

Considering the overall results (row all in Table 7), models M9, M1 and M10 provided the lowest mean relative RMSE values for the recycled cigarette butt, foam and mineral wool samples, respectively. However, these overall results should be interpreted with caution, since, as drawn in the previous section, sample thickness plays a more important role than expected in determining the suitability of each empirical formulation.

### 3.4. Application of the Different Proposed Theoretical Models to the Samples: Combined Spectra

**Figure 8:**
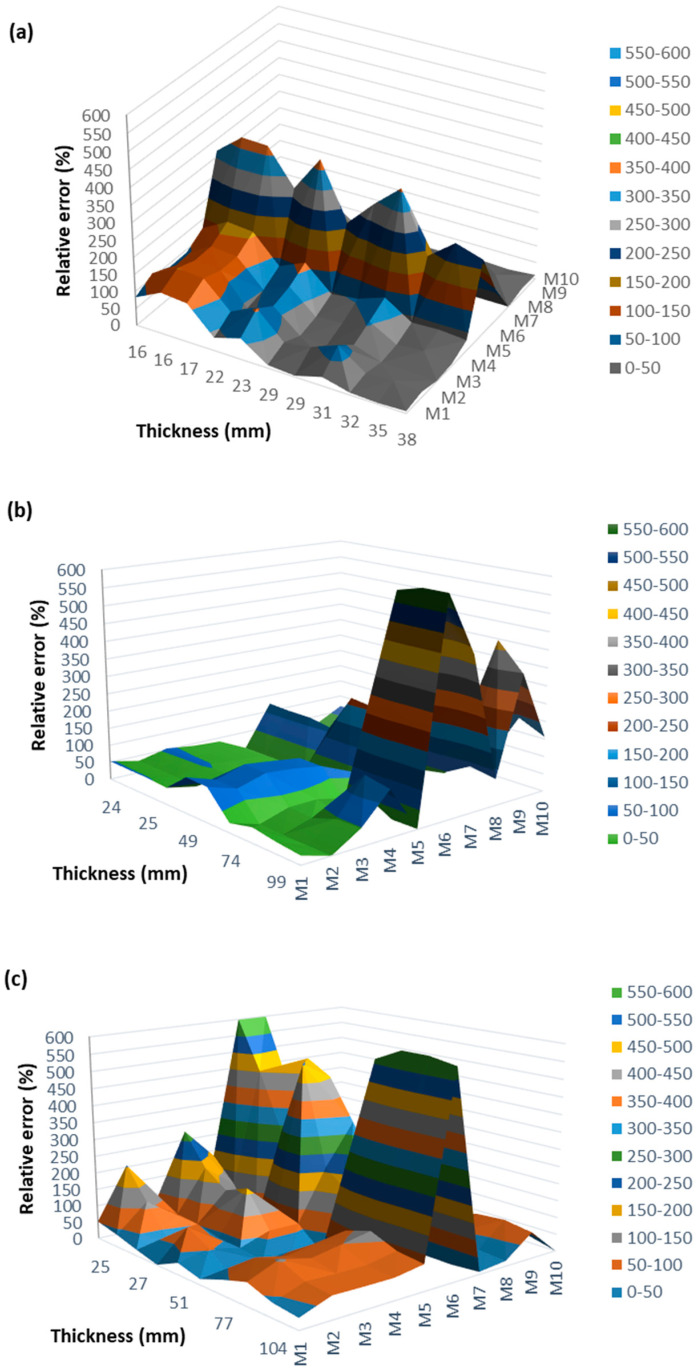
Relative RMSE values for combined samples [(**a**) used cigarette butts; (**b**) foam; (**c**) mineral wool].

The results obtained from the combined spectra, covering the frequency range from 100 to 6400 Hz, are summarized in Table 8. The corresponding relative RMSE values are presented in Figure 8, whereas the mean relative RMSE values, grouped according to material type and sample thickness, are summarized in Table 9.

**Figure 9:**
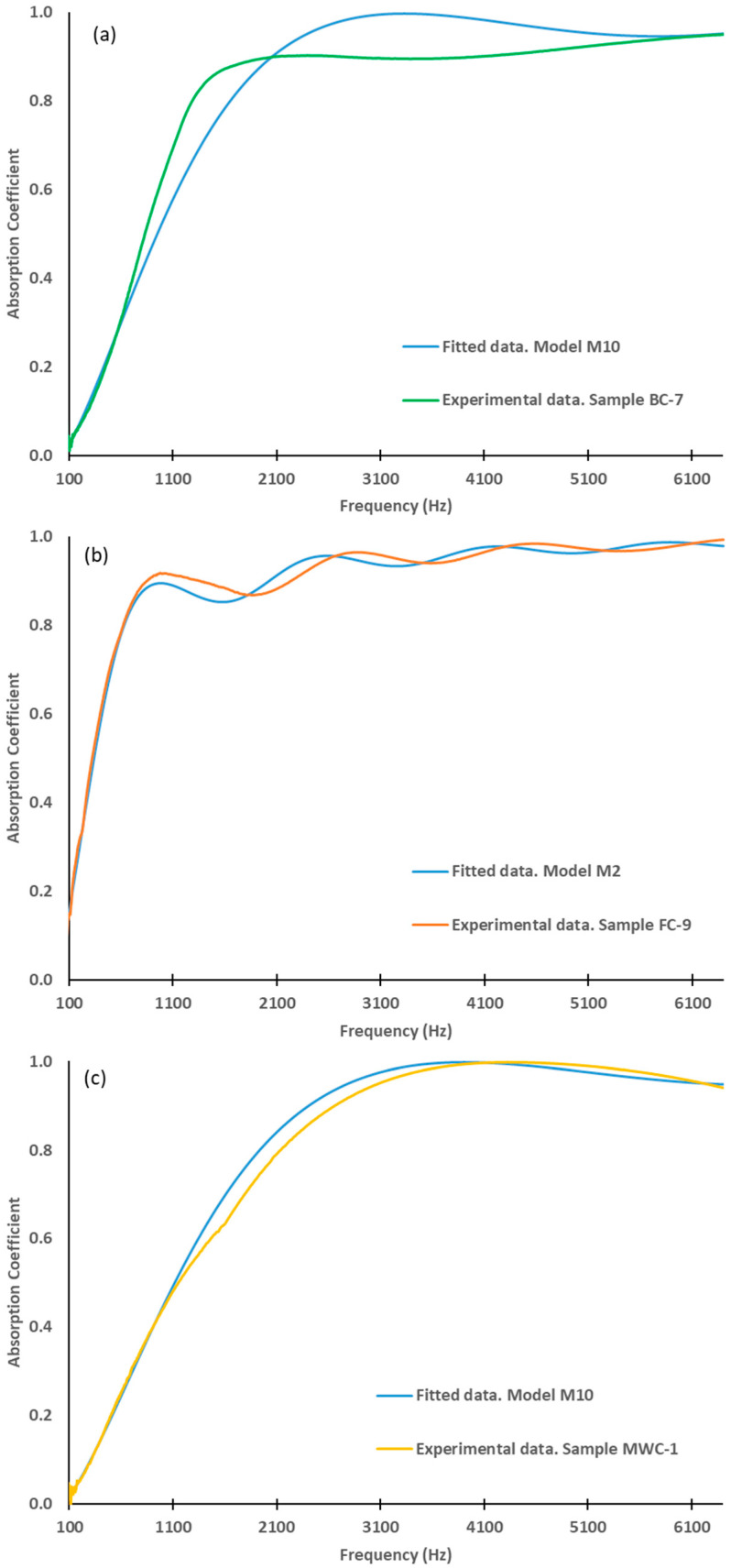
Measured spectra and fitted data for the minimum RMSE values calculated for combined spectra. [(**a**) used cigarette butts; (**b**) foam; (**c**) mineral wool].

For the recycled cigarette butt samples, model M9 yielded the lowest RMSE values for the thinnest samples, whereas models M8 and M10 provided the best agreement as sample thickness increased. The minimum RMSE value was obtained with model M10 for sample BC7 [Figure 9a].

For the foam samples, the optimal model also depends on sample thickness. Model M6 consistently performed best for samples thinner than approximately 50 mm, whereas models M9 and, particularly, M2 provided the lowest RMSE values for thicker samples. The minimum RMSE value was obtained with model M2 for sample FC9 [Figure 9b].

The mineral wool samples exhibited more complex behavior. Models M5, M8 and M10 yielded the lowest RMSE values for the thinnest samples, whereas models M2 and M10 performed best for intermediate thicknesses (50–70 mm). For samples thicker than 70 mm, models M10 and, particularly, M7 provided the best agreement. The minimum RMSE value was obtained with model M10 for sample MWC-1 [Figure 9c].

The analysis of the mean relative RMSE values (Table 9) confirms the strong influence of sample thickness on model performance. For the recycled cigarette butt samples, model M9 clearly outperformed the remaining formulations for thicknesses below 20 mm, whereas several models provided comparable results for thicker samples, with the exception of models M5 and M6, which consistently exhibited poorer performance. Interestingly, model M8, which showed limited accuracy for the individual 29 mm and 100 mm measurements in the 30–40 mm thickness range, provided the best overall performance for the corresponding combined spectra.

For the foam samples, model M6 yielded the lowest mean relative RMSE values for thicknesses below 60 mm, whereas model M2 consistently performed best for thicker samples. Model M5 also provided competitive results for samples between 80 and 100 mm thick.

For the mineral wool samples, models M10 and M1 performed best for the thinnest samples (0–25 mm and 25–50 mm, respectively), model M8 yielded the lowest mean relative RMSE values for samples between 50 and 75 mm thick, and model M7 clearly outperformed the remaining models for samples in the two intervals thicker than 75 mm.

The combined spectra confirm once again that model selection cannot be based solely on the type of material, as it depends on the thickness of the sample.

### 3.5. Comparison of the Results Obtained for the Different Spectrum Types

In the previous section, the results obtained for the three spectrum types (100 mm spectra, 29 mm spectra, and combined spectra) were presented. Table 10 compares the RMSE values obtained for each spectrum type, material, and thickness range.

As shown in Table 10, for recycled cigarette butt and foam samples, the lowest RMSE values for the thinnest samples (<20 and 20–40 mm, respectively) were generally obtained using the 100 mm spectra for almost all models, with the exception of model M9 for cigarette butt samples and model M6 for foam samples.

The combined spectra only provided better results in isolated cases for cigarette butt samples. In contrast, they outperformed the other spectrum types in almost all thickness ranges for foam samples, and for most models within the 25–50 mm thickness range for mineral wool samples.

The models yielding the lowest RMSE values are consistent with those identified in the previous section. For recycled cigarette butt samples, model M9 most frequently produced the lowest RMSE values (five cases), while the remaining minimum values were obtained with models M4, M7, and M8 (one, one, and two cases, respectively).

For foam samples, model M6 clearly provided the best performance in the first two thickness intervals (20–40 and 40–60 mm). For thicker samples, model M2 most frequently produced the lowest RMSE values (three cases), while the remaining minimum values were shared among models M6, M8, and M9 (one case each).

Finally, for mineral wool samples, model M10 produced the lowest RMSE values for the thinnest samples (0–25 mm and 25–50 mm ranges), accounting for three cases. The remaining cases were obtained with models M1 and M2 (one case each). For the thicker samples, the minimum RMSE values were distributed among models M5 (one case), M7 (three cases), M9 (two cases), and M10 (three cases).

## 4. Conclusions

The main conclusions of the present study can be summarized as follows:

Increasing sample thickness significantly modifies not only the magnitude of the sound absorption coefficient but also the overall shape of the sound absorption spectrum. As a consequence, the most suitable empirical model depends not only on the porous material but also on sample thickness and the frequency range considered. Thus, no single model consistently provided the best agreement over the entire thickness range for any of the materials investigated.

The models yielding the lowest mean relative RMSE values for each material, frequency range and thickness interval are summarized in Table 11.

Foam samples exhibited a behavior clearly different from that of the fibrous materials investigated. Models M2 and, particularly, M6 provided the best agreement for most of the foam samples, whereas model M6 consistently performed poorly for the fibrous materials. This result is noteworthy because model M6 was originally proposed for kenaf fiber materials, highlighting that the suitability of these empirical models cannot be inferred solely from the material for which they were originally developed.

For the recycled cigarette butt samples, model M9 provided the best overall performance for the 100 mm impedance tube and for the thinner specimens analyzed using the 29 mm tube and the combined spectra. This agreement is particularly interesting because cigarette filters are composed primarily of cellulose acetate, whereas model M9 was originally developed for cellulose-based materials.

For the mineral wool samples, the most suitable empirical model depended strongly on both sample thickness and frequency range. Model M9 generally provided the best agreement in the 100–1600 Hz frequency range, whereas models M1, M7, M8 and M10 became more suitable for the higher-frequency analyses depending on the sample thickness.

Overall, the present results are consistent with recent validation studies while extending their practical implications. Recent investigations have shown that the prediction accuracy of Delany–Bazley-type empirical models depends on the porous material considered and that no single formulation provides uniformly accurate predictions for all absorber types. Consequently, previous authors have generally recommended either material-specific empirical models or the calibration of model coefficients for the material under investigation [3,33]. Likewise, the comprehensive review by Devi et al. highlighted that different empirical and semi-phenomenological models exhibit different levels of accuracy depending on the porous medium and recommended validating model predictions against experimental measurements whenever possible.

The present study confirms these observations for fibrous absorbers, where the best-performing formulations generally correspond to models originally developed for fibrous materials. However, the systematic comparison performed here also reveals that material type alone is insufficient to identify the most appropriate empirical model due to the influence of thickness. Model M6, originally proposed for kenaf fiber materials, provided the best overall performance for foam samples, whereas model M9 yielded the lowest prediction errors for recycled cigarette butt absorbers despite having been developed for cellulose-based materials.

Therefore, Delany–Bazley-type empirical models should not be selected solely according to the material category for which they were originally proposed. Instead, both the material characteristics, sample thickness, and the frequency range should be considered, with the overall shape of the measured sound absorption spectrum serving as a practical criterion for model selection.

The novelty of the present work does not lie in the development of a new empirical model, but in the systematic comparison of ten Delany–Bazley-based formulations across different porous materials, sample thicknesses and frequency ranges and impedance tube configurations. This comparison provides a practical framework for selecting the most appropriate empirical model for both conventional and recycled porous absorbers under different experimental conditions.

Finally, further studies involving additional porous materials with different microstructural characteristics would be valuable to assess the generality of the trends identified in the present work.

## Figures and Tables

**Table 1:** Number of samples and range of thicknesses of the samples measured.

Diameter of the Samples	Type of Sample	Number of Samples	Range of Thicknesses (mm)
29 mm	Used cigarette butts	15	8.8–38.1
29 mm	Foam	10	23.9–97.9
29 mm	Mineral wool	20	9.7–106.1
100 mm	Used cigarette butts	15	14.0–38.2
100 mm	Foam	10	24.8–99.8
100 mm	Mineral wool	10	26.2–112.8

**Table 3:** RMSE values calculated for each model using the measurements performed with the 100 mm impedance tube. Shaded cells indicate the lowest RMSE value for each sample. B, F and MW denote samples made from recycled cigarette butts, foam and mineral wool, respectively.

Sample	Thickness (mm)	M1	M2	M3	M4	M5	M6	M7	M8	M9	M10
B100-01	14.04	0.100	0.116	0.126	0.138	0.143	0.254	0.077	0.086	0.142	0.097
B100-02	16.05	0.110	0.127	0.138	0.151	0.157	0.279	0.078	0.093	0.169	0.104
B100-03	16.29	0.089	0.107	0.117	0.130	0.137	0.267	0.091	0.074	0.177	0.083
B100-04	17.78	0.189	0.210	0.216	0.234	0.243	0.401	0.127	0.165	0.050	0.179
B100-05	23.33	0.112	0.139	0.136	0.156	0.174	0.382	0.169	0.093	0.149	0.087
B100-06	24.58	0.134	0.158	0.169	0.179	0.192	0.365	0.123	0.118	0.259	0.110
B100-07	25.51	0.178	0.208	0.187	0.213	0.243	0.466	0.207	0.144	0.014	0.144
B100-08	25.53	0.183	0.214	0.204	0.226	0.250	0.478	0.176	0.154	0.060	0.152
B100-09	29.06	0.207	0.241	0.207	0.230	0.273	0.495	0.216	0.169	0.070	0.165
B100-10	29.89	0.142	0.178	0.142	0.159	0.206	0.426	0.258	0.117	0.014	0.100
B100-11	30.48	0.105	0.140	0.116	0.131	0.170	0.420	0.238	0.092	0.120	0.062
B100-12	31.15	0.250	0.285	0.244	0.264	0.314	0.523	0.205	0.208	0.146	0.207
B100-13	32.04	0.218	0.255	0.229	0.246	0.288	0.545	0.198	0.185	0.013	0.170
B100-14	33.99	0.232	0.270	0.227	0.241	0.297	0.516	0.214	0.195	0.118	0.186
B100-15	38.21	0.239	0.278	0.232	0.243	0.303	0.530	0.203	0.202	0.123	0.189
F100-1	24.75	0.247	0.221	0.259	0.257	0.201	0.105	0.702	0.293	0.210	0.279
F100-2	24.86	0.231	0.204	0.242	0.241	0.185	0.088	0.686	0.276	0.194	0.276
F100-3	25.07	0.249	0.222	0.261	0.260	0.203	0.105	0.700	0.294	0.213	0.281
F100-4	25.15	0.259	0.231	0.270	0.269	0.212	0.115	0.710	0.303	0.222	0.291
F100-5	49.61	0.235	0.196	0.245	0.261	0.195	0.062	0.509	0.254	0.224	0.266
F100-6	50.22	0.257	0.218	0.267	0.283	0.217	0.083	0.525	0.276	0.243	0.288
F100-7	74.76	0.148	0.108	0.156	0.153	0.115	0.059	0.361	0.168	0.134	0.180
F100-8	74.97	0.152	0.112	0.160	0.157	0.119	0.058	0.363	0.173	0.137	0.184
F100-9	99.83	0.078	0.036	0.077	0.072	0.047	0.093	0.259	0.097	0.115	0.112
F100-10	99.83	0.083	0.041	0.082	0.076	0.052	0.090	0.263	0.101	0.113	0.116
MW100-1	26.16	0.054	0.084	0.042	0.053	0.105	0.234	0.407	0.062	0.047	0.020
MW100-2	28.32	0.057	0.038	0.072	0.066	0.007	0.127	0.497	0.119	0.064	0.095
MW100-3	28.51	0.116	0.147	0.103	0.109	0.168	0.297	0.369	0.094	0.117	0.079
MW100-4	29.81	0.077	0.112	0.066	0.074	0.132	0.288	0.360	0.072	0.047	0.039
MW100-5	54.67	0.230	0.270	0.222	0.230	0.288	0.515	0.169	0.193	0.163	0.179
MW100-6	58.13	0.195	0.236	0.186	0.193	0.251	0.475	0.177	0.161	0.151	0.143
MW100-7	82.99	0.222	0.264	0.225	0.240	0.277	0.528	0.142	0.188	0.156	0.173
MW100-8	84.29	0.235	0.275	0.238	0.254	0.289	0.536	0.136	0.200	0.162	0.191
MW100-9	112.8	0.224	0.268	0.244	0.262	0.281	0.572	0.141	0.195	0.132	0.176
MW100-10	112.8	0.222	0.266	0.242	0.259	0.279	0.571	0.144	0.193	0.131	0.171

**Table 4:** Mean relative RMSE values for each model and material type obtained from measurements performed with the 100 mm impedance tube, grouped according to sample thickness. Shaded cells indicate the lowest mean relative RMSE value for each material/thickness group. B, F and MW denote samples made from recycled cigarette butts, foam and mineral wool, respectively.

Type	Thickness Interval (mm)	M1	M2	M3	M4	M5	M6	M7	M8	M9	M10
	All	319.2	396.4	345.9	392.0	474.2	1001.5	378.5	250.3	42.8	233.3
B	10–20	91.6	118.9	131.7	153.7	164.0	361.8	43.7	64.4	85.5	81.9
	20–30	420.1	523.9	446.2	514.9	628.2	1334.2	602.6	327.1	34.4	302.7
	30–40	380.0	465.5	396.9	435.1	537.5	1114.0	377.4	307.0	18.8	271.2
	All	158.1	101.1	167.8	167.6	101.4	27.6	578.1	197.5	152.7	209.1
	20–40	139.9	113.5	151.3	149.8	94.9	0.0	582.9	183.9	104.0	174.9
F	40–60	244.2	189.5	258.7	281.0	187.6	0.0	626.8	270.5	227.2	288.1
	60–80	157.1	89.2	170.5	165.3	100.7	0.0	519.6	192.0	131.6	211.4
	80–100	109.3	0.0	107.4	92.2	28.9	138.0	578.1	157.4	196.6	196.4
	All	135.4	154.3	147.0	153.8	136.5	532.3	994.1	205.8	102.9	134.5
	25–50	251.8	254.5	275.0	277.6	194.9	909.7	2473.9	463.1	248.6	302.2
MW	50–75	38.8	65.8	33.5	38.0	76.3	224.7	13.9	15.7	2.9	5.1
	75–100	64.5	93.6	66.6	77.5	103.5	282.4	0.0	39.4	14.2	30.7
	100–125	69.9	103.0	85.1	98.3	112.9	335.1	8.8	47.7	0.0	32.1
All	All	220.6	242.9	238.2	259.8	271.2	589.2	611.4	222.5	91.4	198.2

**Table 5:** Mean relative RMSE for the mineral wool samples measured using the 100 mm impedance tube after excluding sample MW100-2. Results are grouped according to sample thickness. Shaded cells indicate the lowest mean relative RMSE value for each thickness interval.

Type	Thickness Interval (mm)	M1	M2	M3	M4	M5	M6	M7	M8	M9	M10
	All	73.3	124.1	64.6	80.5	151.7	408.2	353.2	57.6	26.7	15.1
	25–50	104.6	197.3	70.3	98.9	259.9	663.2	1044.6	104.4	68.6	0.0
MW	50–75	38.8	65.8	33.5	38.0	76.3	224.7	13.9	15.7	2.9	5.1
	75–100	64.5	93.6	66.6	77.5	103.5	282.4	0.0	39.4	14.2	30.7
	100–125	69.9	103.0	85.1	98.3	112.9	335.1	8.8	47.7	0.0	32.1

**Table 6:** RMSE values calculated for each model using the measurements performed with the 29 mm impedance tube. Shaded cells indicate the lowest RMSE value for each sample. B, F and MW denote samples made from recycled cigarette butts, foam and mineral wool, respectively.

Sample	Thickness (mm)	M1	M2	M3	M4	M5	M6	M7	M8	M9	M10
B29-1	8.78	0.341	0.379	0.360	0.375	0.415	0.690	0.227	0.303	0.058	0.287
B29-2	9.48	0.313	0.353	0.331	0.342	0.386	0.670	0.216	0.275	0.032	0.253
B29-3	10.75	0.319	0.362	0.331	0.339	0.392	0.683	0.214	0.279	0.083	0.254
B29-4	16.04	0.195	0.236	0.188	0.195	0.250	0.469	0.156	0.162	0.162	0.147
B29-5	17.71	0.199	0.240	0.196	0.204	0.255	0.500	0.143	0.164	0.144	0.148
B29-6	18.45	0.181	0.220	0.175	0.185	0.233	0.454	0.130	0.149	0.144	0.140
B29-7	20.97	0.147	0.171	0.138	0.146	0.184	0.309	0.122	0.125	0.163	0.129
B29-8	21.56	0.132	0.159	0.124	0.135	0.170	0.309	0.125	0.110	0.144	0.113
B29-9	22.31	0.129	0.156	0.121	0.134	0.168	0.313	0.118	0.108	0.134	0.113
B29-10	28.37	0.101	0.107	0.097	0.094	0.120	0.200	0.130	0.092	0.122	0.098
B29-11	28.67	0.074	0.087	0.071	0.075	0.097	0.198	0.139	0.069	0.107	0.072
B29-12	30.37	0.083	0.103	0.080	0.088	0.111	0.226	0.115	0.075	0.120	0.074
B29-13	33.46	0.084	0.081	0.083	0.075	0.092	0.164	0.138	0.275	0.104	0.083
B29-14	36.39	0.065	0.061	0.066	0.065	0.070	0.149	0.137	0.303	0.092	0.065
B29-15	38.06	0.076	0.071	0.074	0.066	0.080	0.165	0.123	0.279	0.090	0.078
F29-1	23.88	0.335	0.294	0.337	0.314	0.357	0.208	0.469	0.424	0.134	0.333
F29-2	23.99	0.331	0.290	0.333	0.309	0.298	0.204	0.465	0.349	0.279	0.364
F29-3	24.63	0.350	0.309	0.351	0.327	0.318	0.227	0.477	0.366	0.297	0.382
F29-4	25.44	0.347	0.306	0.347	0.321	0.315	0.229	0.471	0.362	0.295	0.377
F29-5	48.62	0.207	0.184	0.203	0.195	0.188	0.121	0.266	0.203	0.150	0.211
F29-6	49.32	0.218	0.195	0.214	0.208	0.198	0.126	0.276	0.213	0.157	0.221
F29-7	72.5	0.117	0.098	0.115	0.117	0.101	0.101	0.148	0.112	0.091	0.122
F29-8	73.31	0.120	0.101	0.118	0.120	0.104	0.108	0.151	0.116	0.094	0.125
F29-9	97.94	0.014	0.045	0.045	0.058	0.044	0.489	0.037	0.011	0.116	0.041
F29-10	97.94	0.017	0.047	0.049	0.062	0.046	0.489	0.040	0.018	0.115	0.043
MW29-1	9.69	0.263	0.068	0.105	0.097	0.041	0.235	0.364	0.156	0.308	0.166
MW29-2	10.17	0.071	0.034	0.071	0.067	0.013	0.269	0.332	0.122	0.263	0.133
MW29-3	23.09	0.056	0.103	0.062	0.103	0.097	0.271	0.197	0.049	0.145	0.014
MW29-4	23.6	0.050	0.098	0.058	0.100	0.090	0.262	0.198	0.047	0.143	0.017
MW29-5	24.48	0.032	0.078	0.042	0.086	0.070	0.241	0.198	0.039	0.133	0.029
MW29-6	24.8	0.031	0.061	0.048	0.089	0.047	0.212	0.217	0.055	0.135	0.059
MW29-7	34.65	0.087	0.094	0.104	0.129	0.086	0.258	0.215	0.103	0.142	0.103
MW29-8	34.97	0.062	0.080	0.079	0.111	0.072	0.262	0.189	0.076	0.129	0.073
MW29-9	46.69	0.035	0.073	0.048	0.081	0.069	0.301	0.098	0.032	0.106	0.017
MW29-10	49.28	0.044	0.077	0.062	0.093	0.072	0.311	0.109	0.044	0.116	0.032
MW29-11	56.86	0.028	0.065	0.049	0.074	0.064	0.344	0.070	0.027	0.097	0.021
MW29-12	59.45	0.034	0.070	0.049	0.074	0.069	0.359	0.033	0.027	0.097	0.019
MW29-13	71.49	0.040	0.075	0.066	0.083	0.075	0.433	0.059	0.037	0.110	0.021
MW29-14	72.88	0.040	0.076	0.061	0.076	0.076	0.437	0.040	0.033	0.102	0.020
MW29-15	81.66	0.039	0.075	0.071	0.082	0.076	0.486	0.054	0.035	0.113	0.025
MW29-16	83.05	0.027	0.062	0.060	0.067	0.065	0.487	0.042	0.024	0.107	0.035
MW29-17	95.97	0.033	0.069	0.078	0.073	0.075	0.567	0.059	0.029	0.131	0.039
MW29-18	95.97	0.064	0.101	0.105	0.101	0.106	0.591	0.081	0.055	0.143	0.016
MW29-19	106.14	0.057	0.095	0.111	0.097	0.102	0.634	0.091	0.048	0.161	0.024
MW29-20	106.14	0.035	0.072	0.092	0.076	0.081	0.617	0.074	0.030	0.153	0.045

**Table 7:** Mean relative RMSE values for each model and material type obtained from measurements performed with the 29 mm impedance tube, grouped according to sample thickness. Shaded cells indicate the lowest mean relative RMSE value for each material/thickness group. B, F and MW denote samples made from recycled cigarette butts, foam and mineral wool, respectively.

Type	Thickness Interval (mm)	M1	M2	M3	M4	M5	M6	M7	M8	M9	M10
	All	124.7	153.2	128.2	135.1	176.3	396.2	102.5	162.6	25.8	90.2
B	5–20	291.2	345.6	306.4	320.8	384.1	749.1	170.0	241.0	3.8	215.5
	20–30	15.3	34.1	9.1	15.2	46.2	165.3	33.1	0.4	34.9	4.8
	30–40	11.7	13.6	9.9	6.5	27.4	155.3	88.0	247.8	47.5	9.2
	All	54.6	83.3	100.4	113.7	88.7	715.1	132.4	59.7	166.1	99.8
	20–40	79.8	57.9	80.3	67.5	72.6	13.9	148.6	101.8	24.1	90.2
F	40–60	71.9	53.1	68.7	63.1	56.3	0.0	119.4	68.3	24.2	74.9
	60–80	27.8	7.8	26.0	28.2	10.9	13.0	61.3	23.5	0.0	33.5
	80–100	13.9	240.0	246.4	342.2	231.1	3534.5	183.8	3.2	757.8	210.5
	All	123.0	228.6	196.3	270.0	211.9	1553.7	476.7	120.1	513.5	73.2
	0–25	246.6	265.4	210.6	340.7	200.8	1162.9	1123.7	273.4	816.7	216.6
	25–50	36.7	128.9	82.8	176.8	113.7	775.2	270.9	43.2	242.9	9.6
MW	50–75	75.1	250.7	175.2	274.2	247.2	1822.5	145.2	51.3	395.6	0.0
	75–100	93.7	254.1	262.1	270.3	270.4	2284.5	172.6	70.2	454.3	18.7
	100–125	79.6	222.7	291.2	235.4	253.6	2283.6	218.2	51.6	499.4	25.5
All	All	108.41	171.20	152.29	190.33	172.64	981.47	275.43	120.85	273.73	84.79

**Table 8:** RMSE values calculated for each model using the combined spectra. Shaded cells indicate the lowest RMSE value for each sample. B, F and MW denote samples made from recycled cigarette butts, foam and mineral wool, respectively.

Sample	Thickness (mm)	M1	M2	M3	M4	M5	M6	M7	M8	M9	M10
BC1	16.17	0.275	0.319	0.284	0.287	0.337	0.626	0.216	0.238	0.150	0.203
BC2	16.25	0.310	0.352	0.326	0.328	0.374	0.669	0.237	0.274	0.140	0.241
BC3	16.88	0.300	0.343	0.317	0.317	0.363	0.663	0.240	0.263	0.140	0.224
BC4	22.15	0.157	0.195	0.156	0.170	0.204	0.422	0.153	0.134	0.113	0.123
BC5	23.45	0.157	0.199	0.165	0.179	0.208	0.463	0.156	0.133	0.097	0.114
BC6	28.72	0.093	0.112	0.090	0.101	0.123	0.267	0.153	0.085	0.090	0.092
BC7	29.28	0.070	0.097	0.071	0.086	0.105	0.266	0.166	0.068	0.083	0.068
BC8	31.21	0.092	0.126	0.095	0.113	0.133	0.334	0.141	0.081	0.093	0.073
BC9	32.31	0.088	0.097	0.086	0.092	0.110	0.236	0.164	0.082	0.096	0.089
BC10	35.19	0.071	0.083	0.071	0.082	0.093	0.238	0.166	0.068	0.089	0.072
BC11	38.14	0.082	0.090	0.079	0.082	0.101	0.250	0.149	0.077	0.088	0.086
FC-1	24.37	0.330	0.291	0.331	0.309	0.299	0.216	0.482	0.346	0.274	0.359
FC-2	24.52	0.336	0.297	0.337	0.315	0.305	0.221	0.487	0.352	0.281	0.365
FC-3	24.75	0.334	0.295	0.335	0.313	0.302	0.220	0.485	0.349	0.278	0.361
FC-4	25.26	0.340	0.301	0.340	0.315	0.309	0.231	0.487	0.355	0.284	0.367
FC-5	49.12	0.198	0.171	0.195	0.185	0.175	0.099	0.314	0.201	0.151	0.210
FC-6	49.77	0.206	0.180	0.204	0.196	0.183	0.104	0.321	0.209	0.157	0.218
FC-7	73.63	0.105	0.084	0.104	0.105	0.086	0.128	0.204	0.108	0.083	0.117
FC-8	74.14	0.039	0.027	0.033	0.030	0.037	0.186	0.135	0.040	0.077	0.045
FC-9	98.89	0.024	0.018	0.026	0.044	0.018	0.356	0.107	0.034	0.094	0.052
FC-10	98.89	0.026	0.021	0.031	0.047	0.021	0.352	0.109	0.036	0.092	0.053
MWC-1	24.88	0.040	0.080	0.050	0.099	0.074	0.220	0.212	0.037	0.136	0.026
MWC-2	25.80	0.053	0.084	0.061	0.110	0.083	0.215	0.199	0.039	0.138	0.043
MWC-3	26.56	0.049	0.053	0.064	0.099	0.042	0.160	0.249	0.073	0.129	0.078
MWC-4	27.15	0.037	0.068	0.049	0.096	0.066	0.219	0.200	0.036	0.127	0.044
MWC-5	50.68	0.052	0.060	0.052	0.064	0.063	0.123	0.170	0.041	0.133	0.039
MWC-6	53.71	0.137	0.117	0.144	0.143	0.119	0.299	0.240	0.157	0.147	0.170
MWC-7	77.24	0.109	0.138	0.124	0.135	0.146	0.505	0.076	0.093	0.124	0.083
MWC-8	78.59	0.115	0.141	0.127	0.138	0.150	0.506	0.069	0.098	0.126	0.094
MWC-9	104.39	0.112	0.137	0.134	0.135	0.147	0.602	0.079	0.097	0.150	0.096
MWC-10	104.39	0.119	0.149	0.147	0.147	0.159	0.619	0.097	0.104	0.159	0.090

**Table 9:** Mean relative RMSE values for each model and material type obtained from combined measurements, grouped according to sample thickness. Shaded cells indicate the lowest mean relative RMSE value for each material/thickness group. B, F and MW denote samples made from recycled cigarette butts, foam and mineral wool, respectively.

Type	Thickness Interval (mm)	M1	M2	M3	M4	M5	M6	M7	M8	M9	M10
	All	43.0	71.6	46.0	56.5	84.3	294.5	84.8	28.1	10.4	20.6
B	<20	106.1	136.1	116.1	117.1	150.1	355.9	61.4	80.5	0.0	55.8
	20–30	28.1	62.6	29.4	44.8	73.4	288.7	80.0	14.0	6.9	8.6
	30–40	10.7	32.1	9.9	22.8	45.9	254.2	107.2	2.8	21.8	6.3
	All	52.7	28.3	54.2	65.0	34.3	412.2	237.3	67.6	116.4	92.7
	20–40	51.0	33.5	51.4	41.2	37.0	0.0	118.8	58.0	25.9	63.6
F	40–60	99.4	73.6	96.9	88.4	76.9	0.0	214.2	102.7	52.3	111.3
	60–80	34.3	0.7	24.2	18.9	20.0	318.0	271.3	38.5	91.5	52.2
	80–100	28.0	0.0	47.2	135.4	0.7	1742.8	463.4	80.9	386.5	172.8
	All	34.0	81.3	56.9	115.2	81.1	478.8	251.6	25.9	171.0	23.1
	0–25	51.8	205.1	89.9	276.2	183.0	737.5	709.0	42.7	419.6	0.0
	25–50	18.3	77.4	48.5	160.9	65.3	413.6	453.8	24.8	238.5	40.1
	50–75	25.2	26.6	27.2	42.1	31.7	183.9	219.1	19.6	131.8	22.3
MW	75–100	54.8	92.8	73.3	88.1	103.7	596.7	0.0	32.3	72.6	22.5
	100–125	36.8	68.7	66.4	66.5	81.0	624.1	3.7	18.8	83.0	10.4
All	All	43.25	60.76	52.17	78.20	67.16	391.90	187.81	40.10	96.41	44.66

**Table 10:** Mean RMSE values for each model and material type according to sample thickness. Shaded cells indicate the lowest mean RMSE value for each model within each material/thickness group. Bold values indicate the lowest RMSE obtained for each spectrum type. B, F and MW denote samples made from recycled cigarette butts, foam and mineral wool, respectively.

Type	Thickness Interval (mm)		M1	M2	M3	M4	M5	M6	M7	M8	M9	M10
		100 mm	0.122	0.140	0.149	0.163	0.170	0.300	**0.093**	0.104	0.135	0.116
	<20	29 mm	0.258	0.298	0.263	0.273	0.322	0.578	0.181	0.222	**0.104**	0.205
		Combined	0.295	0.338	0.309	0.311	0.358	0.653	0.231	0.258	**0.143**	0.223
		100 mm	0.159	0.190	0.174	0.194	0.223	0.435	0.192	0.133	**0.094**	0.126
B	20–30	29 mm	0.117	0.136	0.110	0.117	0.148	0.266	0.127	**0.101**	0.134	0.105
		Combined	0.119	0.151	0.121	0.134	0.160	0.355	0.157	0.105	**0.096**	0.099
		100 mm	0.209	0.246	0.210	0.225	0.274	0.507	0.212	0.176	**0.104**	0.163
	30–40	29 mm	0.077	0.079	0.076	**0.073**	0.088	0.176	0.128	0.233	0.102	0.075
		Combined	0.083	0.099	0.083	0.092	0.109	0.265	0.155	**0.077**	0.091	0.080
		100 mm	0.246	0.219	0.258	0.257	0.200	**0.103**	0.700	0.291	0.210	0.282
	20–40	29 mm	0.341	0.300	0.342	0.318	0.322	**0.217**	0.470	0.375	0.251	0.364
		Combined	0.335	0.296	0.336	0.313	0.304	**0.222**	0.485	0.350	0.279	0.363
		100 mm	0.246	0.207	0.256	0.272	0.206	**0.072**	0.517	0.265	0.234	0.277
F	40–60	29 mm	0.212	0.189	0.208	0.202	0.193	**0.124**	0.271	0.208	0.154	0.216
		Combined	0.202	0.176	0.199	0.191	0.179	**0.101**	0.318	0.205	0.154	0.214
		100 mm	0.150	0.110	0.158	0.155	0.117	**0.058**	0.362	0.170	0.135	0.182
	60–80	29 mm	0.118	0.100	0.117	0.119	0.103	0.105	0.149	0.114	**0.093**	0.124
		Combined	0.072	**0.056**	0.069	0.068	0.061	0.157	0.170	0.074	0.080	0.081
		100 mm	0.081	**0.039**	0.080	0.074	0.050	0.091	0.261	0.099	0.114	0.114
	80–100	29 mm	0.016	0.046	0.047	0.060	0.045	0.489	0.039	**0.015**	0.115	0.042
		Combined	0.025	**0.019**	0.028	0.045	0.019	0.354	0.108	0.035	0.093	0.052
		100 mm	-	-	-	-	-	-	-	-	-	-
	0–25	29 mm	0.084	0.074	0.064	0.090	**0.060**	0.248	0.251	0.078	0.188	0.070
		Combined	0.040	0.080	0.050	0.099	0.074	0.220	0.212	0.037	0.136	**0.026**
		100 mm	0.076	0.095	0.071	0.076	0.103	0.237	0.408	0.087	0.069	**0.058**
	25–50	29 mm	0.057	0.081	0.073	0.104	0.075	0.283	0.153	0.064	0.123	**0.056**
		Combined	**0.046**	0.068	0.058	0.101	0.064	0.198	0.216	0.049	0.131	0.055
		100 mm	0.212	0.253	0.204	0.211	0.269	0.495	0.173	0.177	**0.157**	0.161
MW	50–75	29 mm	0.036	0.072	0.056	0.077	0.071	0.393	0.051	0.031	0.101	**0.020**
		Combined	0.095	**0.089**	0.098	0.103	0.091	0.211	0.205	0.099	0.140	0.105
		100 mm	0.229	0.269	0.232	0.247	0.283	0.532	**0.139**	0.194	0.159	0.182
	75–100	29 mm	0.041	0.077	0.079	0.081	0.080	0.533	0.059	0.036	0.123	**0.029**
		Combined	0.112	0.140	0.126	0.136	0.148	0.506	**0.073**	0.096	0.125	0.089
		100 mm	0.223	0.267	0.243	0.260	0.280	0.572	0.143	0.194	**0.131**	0.173
	100–125	29 mm	0.046	0.083	0.102	0.087	0.092	0.625	0.083	0.039	0.157	**0.034**
		Combined	0.116	0.143	0.141	0.141	0.153	0.611	**0.088**	0.100	0.155	0.093

**Table 11:** Models with lowest relative RMSE values for each material type according to sample thickness.

Material	Thickness Interval (mm)	100 mm Tube	29 mm Tube	Combined Spectra
Butts	<20	M7	M9	M9
	20–30	M9	M8	M9
	30–40	M9	M4	M8
	20–40	M6	M6	M6
Foam	40–60	M6	M6	M6
	60–80	M6	M9	M2
	80–100	M2	M8	M2
	0–25	--	M5	M10
	25–50	M9	M9	M1
Mineral wool	50–75	M9	M10	M2
	75–100	M7	M10	M7
	100–125	M9	M10	M7

## Data Availability

The original contributions presented in this study are included in the article. Further inquiries can be directed to the corresponding author.
